# Environmental Implications of Nanotechnology—An Update

**DOI:** 10.3390/ijerph8020470

**Published:** 2011-02-10

**Authors:** Leo Stander, Louis Theodore

**Affiliations:** 1 Stander Environmental Engineering, 1117 Fernlea Court, Cary, NC 27511, USA; 2 Theodore Tutorials, 5 Fairview Avenue, East Williston, NY 11596, USA; E-Mail: loutheodore1@verizon.net

**Keywords:** nanotechnology, nanomaterials, nanoparticles, nanoapplications, environmental implications, regulatory compliance, environmental concerns, environmental risk management, risk evaluation process, hazard risk, nano-related regulations

## Abstract

Some engineers and scientists are either directly or indirectly involved with nanotechnology issues. Nanotechnology concerns dealing with environmental implications and regulatory compliance encompass practicing areas for these technical individuals. Areas of particular concern include current/proposed environmental regulations and procedures for quantifying both health risks and hazard risks. This article addresses both of these issues.

## 1. Introduction

Nanotechnology is concerned with the world of miniscule particles that are dominated by forces of physics and chemistry that cannot be applied at the macro- or human-scale level. These particles are defined by some as nanomaterials, and possess unusual properties that are not present in traditional and/or ordinary materials.

Regarding the word *nanotechnology*, it is derived from the words *nano* and *technology. Nano*, typically employed as a prefix, is defined as one-billionth of a quantity or term. It is represented mathematically as 1 × 10^−9^, or simply as 10^−9^. *Technology* generally refers to “the system by which a society provides its members with those things needed or desired.” The term *nanotechnology* has come to be defined as those systems or processes that provide goods and/or services that are obtained from matter at the nanometer level, *i.e.*, from sizes in the range of one-billionth of a meter. In addition, the new technology allows the engineering of matter by systems and/or processes that deal with atoms, or to paraphrase K. E. Drexler, “Nanotechnology is the principle of manipulation of the structure of matter at the molecular level. It entails the ability to build molecular systems with atom-by-atom precision, yielding a variety of nanomachines [1].”

The classic laws of science are different at the nanoscale. Nanoparticles possess large surface areas and essentially no inner mass, *i.e.*, their surface-to-mass ratio is extremely high. The new “science” of nanotechnology is based on the fact that particles in the nanometer range, and nanostructures or nanomachines that are developed from these nanoparticles possess special properties and exhibit unique behavior. These special properties, in conjunction with the unique behavior of nanomaterials, can significantly impact physical, chemical, electrical, biological, mechanical, and functional qualities and properties. These newly identified characteristics can be harnessed and exploited by applied scientists to engineer new processes.

## 2. Environmental Concerns

The authors believe that nanotechnology is the second coming of the Industrial Revolution, or as one of the authors [2] has described it, “Industrial Revolution II.” It promises to make that nation that seizes the nanotechnology initiative the technology capital of the world. One of the main obstacles to achieving this goal will be to control, reduce, and ultimately eliminate environmental and environmentally-related problems associated with this technology. The success or failure of this effort may well depend on the ability to address these environmental issues.

Only time will provide answers to many key environmental questions, including the following:

1. What are the potential environmental concerns associated with this new technology?
2. Can industry and society expect toxic/hazardous material to be released into the environment during either the manufacture or use of nanoproducts?
3. Could nanoapplications lead to environmental degradation, particularly from bioaccumulation of nanoproducts in living tissue?
4. What impact will regulations have on this new technology?

Regarding these questions, the environmental health and hazard risks associated with both nanoparticles and the applications of nanotechnology for commercial and industrial uses are not fully known [2–8]. Some early studies suggest that nanoparticles might serve as environmental poisons that accumulate in organs. Although these risks may prove to be either minor, avoidable, or both, the engineer and scientist are duty bound to determine if there are in fact any health, safety, and environmental impacts associated with nanotechnology [2]. This information is also of vital importance to those involved and engaged in the legal arena.

## 3. Environmental Risk Management

People face all kinds of risk every day, some voluntarily and others involuntarily. Therefore, risk plays a very important role in today’s world. Studies on cancer caused a turning point in the world of risk because it opened the eyes of risk engineers/scientists and health professionals to the world of risk assessments.

Unfortunately, the word risk has come to mean different things to different people. The dictionary defines risk as “the chance of injury, danger or loss… to expose to the chance or injury, damage, or loss.” Stander and Theodore have defined it as “a combination of uncertainty and damage [8].” To compound this problem, there are two types of environmental risks that professionals are concerned with: *health* risk and *hazard* risk. However, these two classes of risk have been used interchangeably by practitioners, researchers, and regulators. Because of this confusion, one of the main objectives of this paper is to both define and clarify the difference between these two risks.

Regarding health risk, concerns arise because substances such as chemicals and nanomaterials can elude defense mechanisms and enter the body. Following exposure, such substances enter the body via various pathways, including inhalation, skin absorption (absorption), and ingestion (digestion system). It is fair to say that a dominant route of entry is inhalation. Note that two types of potential exposures exist [2,8]:

1. *Chronic*: Continuous exposure occurs over longer periods of time, generally months to years. Concentrations of inhaled toxic contaminants are relatively low. Direct skin contact by immersion, splash, or by contaminated air involves contact with substances exhibiting low dermal activity.
2. *Acute*: Exposures occur for relatively short periods of time, generally minutes to 1–2 days. Concentrations of toxic air contaminants are high. In addition to inhalation, airborne substances might directly contact the skin, or liquids and sludges may be splashed on the skin or into the eyes, leading to toxic effects.

Alternatively, hazard risks (*i.e.*, risks of equipment malfunctions, upset conditions, or accidents)— which are classified in the acute category—involve a triple combination of event, probability, and consequences that can provide a measure of economic loss or human injury in terms of both the incident likelihood and the magnitude of the loss or injury. Hazard risk, which is generally concerned with “accidents,” is of vital concern in manufacturing activities and plant operations, and can arise as either a health risk (as described in the previous paragraph) to workers at the facility or others in the surrounding community, or a risk associated with an accident, or both (examples include the many uses of high pressure steam or the operation of an oil drilling facility—either on or off shore).

Virtually all environmental concerns are related directly or indirectly with risk. Any discussion of environmental health and hazard concerns associated with nanomaterials and nanoprocesses must, therefore, also address the issue of risk. The next two sections review traditional and time-tested methods that the practicing engineer and scientist employ in health and hazard risk analysis/assessment—procedures that are employed in the nanotechnology field today.

## 4. The Health Risk Evaluation Process [2,7–9]

As indicated above, many environmental practitioners, researchers, and regulators have confused health risks with hazard risks, and vice versa. Although both employ a multi-step method of analysis, the procedures are quite different, with each providing different results, information, and conclusions. Both share a common concern in that they can negatively impact individuals, society, and the environment. Environmental health risk and the environmental risk assessment processes are widely discussed in technical literature and are the bases of many health, safety, and environmental management activities [2,10–13].

Health risk assessment provides an orderly, explicit and consistent way to deal with scientific issues in evaluating whether a health problem exists and what the magnitude of the problem may be. This evaluation typically involves large uncertainties because the available scientific data is limited, and the mechanisms for adverse health impacts or environmental damage are only imperfectly understood.

**Figure 1.**
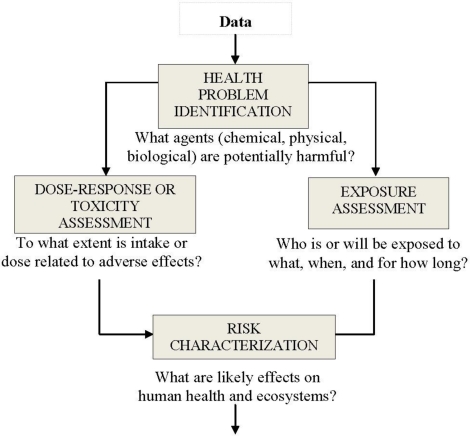
The Health Risk Evaluation Flowchart.

Most human or environmental health problems can be evaluated by dissecting the analysis into four parts: problem identification, dose-response assessment, exposure assessment, and risk characterization (see Figure 1). This four step framework has been widely adopted by U.S federal/state agencies and international organizations that assess and manage health and environmental issues [10–13].

For some perceived problem, the risk assessment might stop with the first step in the process, *i.e.*, problem identification, if no adverse effect is identified or if an agency elects to take regulatory action without further analysis [9]. Regarding health problem identification, a problem may be defined as a toxic agent or a set of conditions that has the potential to cause adverse effects to human health or the environment. Problem identification involves an evaluation of various forms of information in order to identify the different health concerns. Dose-response or toxicity assessment is required in an overall assessment: responses/effects can be different since all chemicals and contaminants vary in their capacity to cause adverse effects. This step frequently requires that assumptions be made to relate experimental data for animals to humans. Exposure assessment is the determination of the magnitude, frequency, duration, and routes of exposure of human populations and ecosystems. Finally, in risk characterization, toxicology and exposure data/information are combined to obtain a qualitative or quantitative expression of risk. Additional details are available in the literature.

With regard to nanomaterials, the health risk evaluation process may be problematic. There is just not enough published data on the environmental health effects resulting from exposure to nanoparticles or protocols or methodologies for making such evaluations [3,14]. To resolve this concern, entities such as the US National Institute of Occupational Safety and Health have issued interim guidance regarding medical screening for workers exposed to engineered nanoparticles [7]. Although some information is available concerning the fates and effects of some classes of nanomaterials in the environment [4], procedures to predict environmental exposures to engineered nanoparticles [5], and techniques that might be used to model environmental concentrations [6], additional information on occupational, consumer, and environmental exposure is needed [3].

## 5. The Hazard Risk Evaluation Process [8,9]

As with environmental health risk, there is a serious lack of information on hazard risks and associated implications of these hazards, particularly with regard to the production and use of nanomaterials [3]. The unknowns in this risk area may be larger in number and greater in potential consequences. It is the authors’ judgment that hazard risks have unfortunately received something less than the attention they deserve. However, hazard risk analysis details are available and traditional approaches have been successfully applied in the past.

In a previous section, both “chronic” and “acute” problems were defined. As indicated, when the two terms are applied to emissions, the former usually refers to ordinary round-the-clock, everyday emissions while the latter term deals with short, out-of-the-norm, accidental emissions. Thus, acute problems normally refer to accidents and/or hazards.

**Figure 2.**
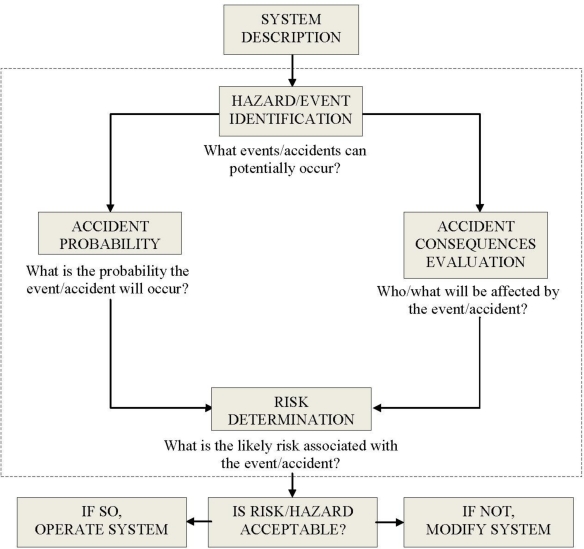
Hazard Risk Assessment Flowchart.

As with assessing environmental health risks of a substance, there are several steps in evaluating the risk of a hazard (including upset conditions, malfunctions, or accidents) at a facility. These are detailed in Figure 2 if the system in question is a chemical plant. The heart of the hazard risk assessment algorithm is enclosed in the dashed box of Figure 2. The algorithm allows for reevaluation of the process if the risk is deemed unacceptable. Similar approaches will likely be utilized in the manufacture of nanomaterials.

## 6. Regulatory Concerns

Many environmental concerns are addressed through existing health and safety legislation. Most countries require a health and safety assessment for any new chemical before it can be marketed. For example, in 2006 the European Union adopted a regulation (EC 1907/2006) on chemicals and their safe use and established the European Chemicals Agency in Helsinki. This Agency manages the **R**egistration, **E**valuation, **A**uthorisation and Restriction of **Ch**emical (REACH) substances system which is a database of information provided by manufacturers and importers on the properties of their chemical substances. Prior experience with materials such as PCBs, dioxins, furans, and, and a variety of unintended effects of drugs such as thalidomide, means that companies and governments have incentives to keep a close watch on potential negative environmental health and hazard effects [15].

It should be noted that there are no nano or nano-related environmental regulations in the US or the EU at this time which require controls on process releases or production activities or specific workplace safety measures. Completely new legislation and regulatory efforts may be necessary to protect the public and the environment from the potential adverse effects of nanotechnology. Until such effects are identified and control or mitigation procedures are developed, one may only speculate on how the existing regulatory framework might be applied as this emerging field develops over the next several years. Detailed analyses of various existing US and EU laws and requirements and similar conclusions are discussed in the literature [16–20].

## 7. Potential Future Legislative and Regulatory Actions in the USA

The principal US agencies concerned with environmental risks are the Environmental Protection Agency (EPA) and the Occupational Safety and Health Administration (OSHA). The mission of EPA is to protect human health and the environment. One of EPA’s major purposes is to ensure that all Americans are protected from significant risks to human health and the environment where they live, learn, and work. The mission of OSHA is to ensure safe and healthful working conditions for working men and women by setting and enforcing standards and by providing training, outreach, education and assistance. Both are therefore directly concerned with environmental implications of nanotechnology.

It is very difficult to predict what future requirements might come into play for nanomaterials. In the past, regulations have been both a moving target and confusing. What can be said is that there will be regulations and the probability is high that they will be contradictory and confusing. Past and current regulations provide a measure of what can be expected. Control of the production and use of nanomaterials is most likely to occur under the Clean Air Act (CAA) and the Toxic Substances Control Act (TSCA) as discussed below both of which are concerned with environmental health impacts.

Under the Clean Air Act no specific requirements or regulatory procedures currently exist for nanoparticles. The use and production of nanomaterials could be regulated in the following circumstances (neither of which is under consideration at this time):

1. An installation that manufactures or uses nanomaterials may become subject to requirements of State Implementation Plans which were developed to assure attainment and maintenance of National Ambient Air Quality Standards for criteria pollutants (including particulate matter with a diameter that is smaller than 2.5 μm (PM_2.5_)). While emissions of nanoparticles may not be specifically subject to the various requirements, the processes involved with producing such substances may result in emissions of criteria pollutants which must be controlled.
2. An installation that is using or manufacturing nanomaterials may become subject to the requirements of Section 112 of the Clean Air Act should such materials become identified as hazardous air pollutants. The Clean Air Act provides a list of 189 substances that have been determined to be hazardous air pollutants. The Act also prescribes procedures for adding and deleting substances from this list. If adverse health and environmental effects are encountered as a result of emissions from the use or manufacture of nanomaterials, the EPA will be forced to list such substances as hazardous air pollutants and require emission controls.

Commercial applications of nanotechnology are more likely to be regulated under TSCA, which authorizes the EPA to review and establish limits on the manufacture, processing, distribution, use, and/or disposal of new materials that pose an unreasonable risk of injury to human health or the environment. The term “chemical” is defined broadly under TSCA. Unless a particular nanomaterial qualifies for an exemption under the law, a prospective manufacturer with low-volume production, with low-level environmental releases along with low volume, or with plans for limited test marketing would be subject to the full evaluation procedures. As previously indicated, this would include a submittal of a premanufacturing notice, along with toxicity and other data, to EPA at least 90 days prior to commencing production of the substance, followed by required recordkeeping and reporting. Requirements will differ, depending on whether EPA determines that a particular application constitutes a “significant new use” or a “new chemical substance.” The EPA can impose limits on production, including an outright ban when it is deemed necessary for adequate protection against “an unreasonable risk of injury to health or the environment.” The EPA may revisit a chemical’s status under TSCA and change the degree or type of regulation when new health/environmental data warrant [21–23]. If the experience with genetically engineered organisms is any indication, there will probably be a push for not only EPA but also OSHA to update regulations in the future to reflect changes, advances, and trends in nanotechnology.

The future of nanotechnology in not known. Scientists, engineers, and even manufacturers can only speculate on its implications or the magnitude of its impact on environmental health. What some might view as a learned prediction of what the future will bring, others might consider as science fiction. The same is true with regard to future legal and regulatory approaches to managing environmental health and hazard risks. As previously discussed, many environmental scientists, attorneys, and others have speculated and offered their perspectives on future regulatory activities [16,17]. Others offer alternative view points. One of the authors of this article has speculated on the need for future regulations for nanomaterials. His suggestions and potential options are available in the literature [20], noting that the ratio of nanoparticles that are currently being emitted from conventional sources such as power plants to present-day engineered nanoparticles being released into the environment may be as high as a trillion to one (*i.e.*, 1 × 10^12^:1 or more simply 10^12^:1) [24]. If this ratio is correct, the environmental concerns associated with today’s nanoparticles can almost certainly be dismissed.
